# How are growth hormone and insulin-like growth factor-1 reported as markers for drug effectiveness in clinical acromegaly research? A comprehensive methodologic review

**DOI:** 10.1007/s11102-018-0884-4

**Published:** 2018-03-31

**Authors:** Michiel J. van Esdonk, Eline J. M. van Zutphen, Ferdinand Roelfsema, Alberto M. Pereira, Piet H. van der Graaf, Nienke R. Biermasz, Jasper Stevens, Jacobus Burggraaf

**Affiliations:** 1 Division of Systems Biomedicine and Pharmacology, Leiden Academic Centre for Drug Research, Leiden University, Leiden, The Netherlands; 2 Centre for Human Drug Research, Leiden, The Netherlands; 3 Division of Endocrinology, Department of Medicine, Leiden University Medical Center, Leiden, The Netherlands; 4 Certara QSP, Canterbury, UK; 5 Department of Clinical Pharmacy and Pharmacology, University Medical Center Groningen, University of Groningen, Groningen, The Netherlands

**Keywords:** Acromegaly, Growth hormone, Pituitary adenoma, IGF-1—review

## Abstract

**Objective:**

In rare disease research, most randomized prospective clinical trials can only use limited number of patients and are comprised of highly heterogeneous populations. Therefore, it is crucial to report the results in such a manner that it allows for comparison of treatment effectiveness and biochemical control between studies. The aim of this review was to investigate the current methods that are being applied to measure and report growth hormone (GH) and insulin-like growth factor-1 (IGF-1) as markers for drug effectiveness in clinical acromegaly research.

**Search strategy:**

A systematic search of recent prospective and retrospective studies, published between 2012 and 2017, that studied the effects of somatostatin analogues or dopamine agonists in acromegaly patients was performed. The markers of interest were GH, IGF-1, and the suppression of GH after an oral glucose tolerance test (OGTT). Additionally, the use of pharmacokinetic (PK) measurements in these studies was analyzed. The sampling design, cut-off for biochemical control, reported units, and used summary statistics were summarized.

**Results:**

A total of 49 articles were selected out of the 263 screened abstracts. IGF-1 concentrations were measured in all 49 studies, GH in 45 studies, and an OGTT was performed in 11 studies. A wide range of different cut-off values and sampling designs were used to determine biochemical control in acromegaly patients. The summary statistics were reported in various ways, with the percentage of biochemical control most frequently used. Nine studies sampled the PK at one or more time points. Non-compartmental analyses were commonly performed on the available PK data.

**Conclusions:**

The way GH and IGF-1 are measured and reported in acromegaly research varies considerably. A consensus on how to report study results would enable better comparisons between studies, thereby improving evidence based decision making to optimize treatment in acromegaly.

**Electronic supplementary material:**

The online version of this article (10.1007/s11102-018-0884-4) contains supplementary material, which is available to authorized users.

## Introduction

Acromegaly is a rare disease (prevalence of 60–70 per million [1]) characterized by growth hormone (GH) hypersecretion that results in the abnormal growth of extremities, high morbidity, and an increased mortality risk. In virtually all cases, acromegaly is the result of a GH secreting pituitary adenoma [2–4]. Under normal physiological conditions, GH is secreted in discrete bursts that result in a pulsatile plasma GH concentration–time profile. GH secretion is mainly upregulated by growth hormone-releasing hormone (GHRH), GH-releasing peptide (GHRP, e.g. ghrelin), and inhibited by somatostatin. Binding of GH to GH-receptors located in the liver induces insulin-like growth factor-1 (IGF-I) synthesis and secretion into the circulation. Negative feedback on GH secretion is mediated by IGF-1 and by GH itself [5]. Major negative determinants of GH secretion are aging and adiposity, while on the other hand aromatizable sex steroids amplify GH secretion [6, 7].

### Guideline recommendations

Both the GH and IGF-1 plasma concentrations are typically increased in active acromegaly and will decrease during effective treatment. Both biomarkers are therefore used in clinical practice to monitor biochemical control in acromegaly and to determine treatment effectiveness. As a result of the biological mechanisms underlying acromegaly, the most recent guidelines, by the Endocrine Society and the American Association of Clinical Endocrinologists (AACE), for the diagnosis and treatment monitoring in acromegaly focus on three key biomarkers; (a) IGF-1, (b) (mean) GH and (c) level of suppression of GH concentrations during an oral glucose tolerance test (OGTT), further referred to in this article as the biomarkers of interest [8, 9].

#### Insulin-like growth factor-1

In clinical practice, a main treatment goal is a reduction in IGF-1 concentrations to the clinically accepted ‘normal’ values for age and sex, which has been associated with improved/normalized mortality. In the guidelines, the upper limit of normal (ULN) was introduced as a surrogate for ‘safe’ IGF-1 levels which can be used to monitor the biochemical control of an individual acromegaly patient. This ULN is commonly defined by 2 × the standard deviation (SD) of normal values, for age and sex, where age related changes have the largest impact on IGF-1 concentrations [10, 11]. Additionally, ULN corrected values have the added benefit that it can be used as a comparable measure of IGF-1 concentrations between individuals.

#### Growth hormone

The primarily pathologically affected hormone in acromegaly is GH. A random GH measurement is therefore performed to provide an indication of the actual endogenous 24 h GH profile. However, the use of random GH levels, or the mean of multiple samples, to monitor treatment effectiveness has many challenges (e.g. highly pulsatile profile of GH, assay variability, lack of a safe range) but requires minimal clinical effort to obtain, compared to a full 24 h GH profile with short sampling intervals which is not feasible in clinical practice. Therefore, IGF-1 is generally considered as a better and more stable biomarker. The 2014 Endocrine Society guideline suggests the use of a random GH measurement (cut-off < 1 ng/ml), with only a low level of evidence for determining biochemical control [8]. Due to the challenges associated with random GH concentrations, the guidelines reports that these values should be handled with caution [8, 9]. For example, we have recently demonstrated that a single random GH measurement underestimates the actual level of GH secretion in patients treated with somatostatin analogues [12].

#### Oral glucose tolerance test

An OGTT is performed as a test to differentiate between healthy individuals and patients with active acromegaly. Furthermore, an OGTT can be performed already 1 week after surgery to assess successful reduction of GH secretion [13]. In healthy individuals, the increase in plasma glucose levels suppresses GH secretion to well below 1 ng/ml [9]. Insufficient suppression of GH is indicative for disruption in the regulation of the hypothalamus–pituitary–somatotropic axis. A ‘standard’ OGTT is performed using 75 g of orally administered glucose and the monitoring of blood samples for GH concentrations every 30 min for 2 h. At present, the recommended cut-off used both for biochemical control, and the diagnosis of acromegaly, is a nadir GH of < 1 ng/ml [8], which has been re-adapted in 2014 from a previously more sensitive cut-off value of 0.4 ng/ml [9]. However, the quantification of GH concentrations in these lower regions is highly dependent on the used analytical assay.

### Study comparison

There are limited prospective clinical trials that included large cohorts of acromegaly patients, due to the low prevalence of acromegaly. Therefore, systematic reviews and meta-analyses serve as a powerful tool to combine study results, thereby increasing statistical power [14]. For correct comparison of studies, the cut-off values used to determine biochemical control, and thereby the quantification of a response rate of a drug, should preferably be equal. As a complicating factor, the assays used to analyze serum GH and IGF-1 samples are heterogeneous and have been previously identified as important factors of influence [15]. Different international recombinant reference preparations have been used for the analysis of both hormones, which lead to discrepancies in the concentrations of the reported biomarkers between studies [15]. Additionally, the proposed cut-off values reported in the guidelines are not assay specific which results in a bias in the interpretation of the proportion of patients with biochemical control between studies [15].

### Pharmacokinetics

To establish the effectiveness of drug treatment in general, the investigated drug must reach the site of action, e.g. the pituitary for somatostatin analogues and dopamine agonists [16]. Sufficient circulating plasma/serum drug concentrations should be reached and maintained in order to drive the desired drug effect at the level of the pituitary. These concentrations should be within the drug-specific therapeutic window in order to trigger an effect. Commonly, to assess the responsiveness of a patient to octreotide, which is a somatostatin analog, a suppression test is performed with a single dose. However, this test does not take into account the circulating plasma/serum concentrations that are reached and is perhaps not the most reliable predictor of drug effectiveness [17]. Information on the pharmacokinetic (PK) profile of an individual patient can give additional information on the level of inter-individual variability in response to a certain dose. This, in combination with the somatostatin subtype receptor expression of the target tissue could inform endocrinologists on the optimal treatment of patients [18].

### Aim

The aim of this review is to summarize the methods that are being applied to measure and report GH and IGF-1 in studies that evaluated medical treatment efficacy in acromegaly patients in peer-reviewed journals, published between 2012 and 2017. It is assumed that this selection provides a good representation of the current state of the reporting of biomarkers, following the recommended cut-off values proposed in the 2011 guideline [9]. In addition, we made an overview of studies measuring the PK of the drugs of interest and the performed PK analysis. Consequently, this will provide a perspective on the consistencies in the used methodology and reporting of studies in acromegaly research and therewith in the comparability of study results.

## Methods

### In- and exclusion criteria

Prospective and retrospective clinical studies, that included a minimum of five acromegaly patients treated with the standard of treatment of somatostatin analogues (Octreotide, Lanreotide or Pasireotide) or dopamine agonists (Bromocriptine, Cabergoline), and investigated the effect of treatment on one or more biomarkers of interest were included. Studies solely investigating the effect of GH receptor antagonists were excluded, except when a GH receptor antagonist was studied as a separate cohort or in combination therapy with a somatostatin analog or dopamine agonist. Review articles, case studies, in vitro experiments and experiments in non-human species were excluded.

### Search strategies

Identification of relevant studies was done using the MEDLINE database accessed through PubMed. Electronic search was performed on October 18th 2017. Studies which were indexed between January 2012 and October 2017, up until the search date, were included in this review. All included studies were therefore published after the release of the 2011 guideline [9]. Sensitive search in PubMed was done using the search terms #1 to #5 presented below, where *CompoundName* was replaced by the expanded names of Bromocriptine, Cabergoline, Octreotide, Lanreotide and Pasireotide. The full search term can be found in the Online Resource 1. Search results were extracted from PubMed in plain text format.

*#1(CompoundName [MeSH Terms*/*tiab])*

*#2 AND (Acromegaly [MeSH Terms] OR Acromegaly [tiab] OR Somatotropinoma [tiab] OR pituitary adenoma [MeSH Terms] OR pituitary adenoma [tiab])*

*#3 NOT Prolactinoma [tiab]*

*#4 NOT (Review [Publication Type] OR Case reports [Publication Type])*

*#5 AND (“2012*/*01*/*01″[Date - Publication] : “2018″[Date - Publication])*

### Study selection and data extraction

All studies were screened on title, abstract, and keywords. For selected studies, full text articles were studied and checked against the in- and exclusion criteria. For all included full text articles, study characteristics were summarized in a data extraction form. This form consisted of general study characteristics, information on the study design, the used biomarkers, analytical assay, and the reported summary statistics. An overview of all variables documented in this data extraction form can be found in Online Resource 2.

## Results

**Fig. 1:**
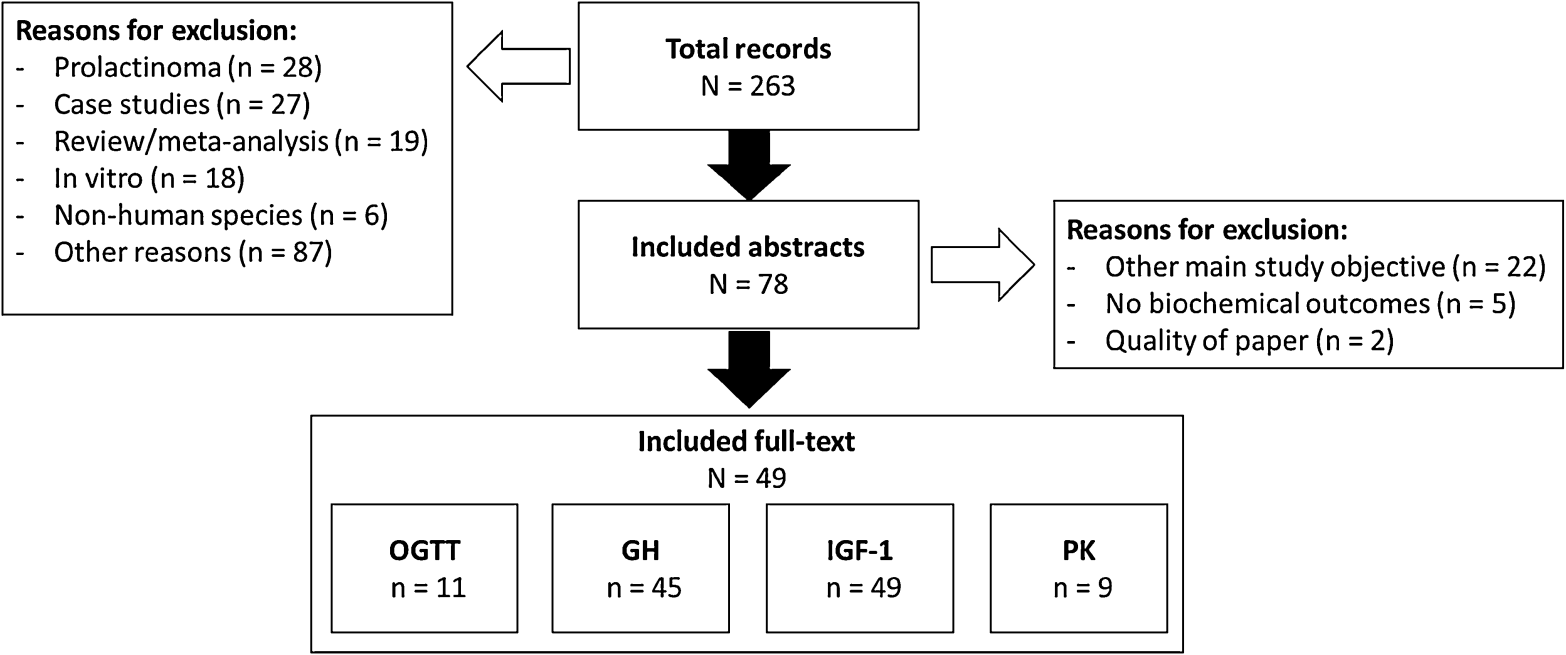
Flow diagram of included studies and main reasons for exclusion. *N* total number, *n* subset of total, *OGTT* oral glucose tolerance test, *GH* growth hormone, *IGF-1* insulin-like growth factor 1, *PK* pharmacokinetics

A total of 49 studies fulfilled all in- and exclusion criteria (Fig. 1). The selected articles cover a wide range of study designs, from randomized phase I clinical trials to post-marketing approval retrospective database analyses. The majority of articles reported on prospective trials (63%). In total, the studies report on data of more than 6400 patients. The median number of subjects that completed a study was 58, with a 25–75% interquartile range [IQR] from 27 to 107. One article included a large number of patients: 2572 patients from the United Kingdom that were included in a retrospective database analysis [19]. The patient populations in the studies were diverse, varying from treatment naïve to long term somatostatin treatment after surgery. An extensive overview of the used study designs and the patient populations per study has been included in Online Resource 3.

The IGF-1 concentrations were measured in all 49 studies (Table 1), GH was measured in 45 studies (Table 2), and an OGTT was performed in 11 studies (Table 3). The tables present a summary of the reporting of the sampling design, used cut-offs, used summary statistics and the frequency of reporting the analytical assay for each biomarker. Approximately 92% of the studies reported both the IGF-1 and the GH response in the population. One article was excluded from the summary tables due to the reporting of a population pharmacokinetic/pharmacodynamic (PK/PD) model instead of focusing on the biochemical control in the population, which will therefore be reported in the “Pharmacokinetics” section [20]. Only nine studies measured and reported the PK of the drug of interest (Table 4).

**Table 1:** Overview of the methods, cut-off and statistical reporting of insulin-like growth factor 1 observations (n = 48), ordered by frequency

IGF-1 analysis	Number of studies (%)	References
Method		
1 Fasting sample	13 (27%)	[21–33]
1 Sample	12 (25%)	[34–45]
Mean of 2 samples (30 min and 1 min before drug administration)	1 (2%)	[46]
Not reported	22 (46%)	[19, 47–67]
Reported units		
ULN corrected	29 (60%)	[21–28, 30–32, 34, 37–39, 41–43, 45, 48–50, 52–55, 59, 65, 66]
ng/ml	21 (44%)	[21, 22, 29, 33, 35, 36, 40, 44, 46, 47, 49, 51, 56, 58–60, 63–67]
SD-score	2 (4%)	[44, 63]
ng/dl	1 (2%)	[31]
nmol/L	1 (2%)	[57]
Not reported	3 (6%)	[19, 61, 62]
IGF-1 cut-off for biochemical control		
< ULN	41 (85%)	[22–27, 29, 31, 33–44, 46–67]
< 1.2x ULN	6 (13%)	[21, 28, 30, 36, 45, 54]
< 1.3x ULN	2 (4%)	[31, 34]
< 1.1x ULN	1 (2%)	[32]
< 1.5x ULN	1 (2%)	[26]
> 20% decrease from baseline	1 (2%)	[21]
> 50% decrease from baseline	1 (2%)	[28]
No cut-off reported/used	1 (2%)	[19]
Summary statistics		
% Biochemical control	41 (85%)	[19, 21–31, 33–35, 39, 41–48, 50–63, 65–67]
Mean ± SD	20 (42%)	[23, 24, 26, 29, 33–35, 38, 39, 41, 42, 44, 46, 48, 54, 55, 57, 63, 66, 67]
Individual ULN corrected levels	17 (35%)	[21, 24–26, 30–32, 37, 38, 41, 44, 45, 48, 53, 54, 63, 65]
% Change mean ± SD	10 (21%)	[22, 24, 30, 35, 46, 48, 57, 63, 65, 67]
Median (range)	10 (21%)	[31, 37, 42, 48, 51–54, 57, 64]
Median (IQR)	9 (19%)	[21, 24, 28, 29, 34, 45, 48, 49, 59]
% Biochemical control [95% CI] no method reported	8 (17%)	[23, 34, 36, 39, 42–44, 46]
Mean ± SD [range]	5 (10%)	[30, 47, 54, 60, 65]
Mean ± SE	5 (10%)	[22, 29, 36, 43, 55]
% Change individual concentrations	4 (8%)	[21, 31, 53, 65]
Mean	4 (8%)	[31, 39, 43, 47]
% Change median	3 (6%)	[22, 37, 64]
% Change median [range]	3 (6%)	[37, 52, 57]
% Change median [IQR]	3 (6%)	[32, 40, 49]
Individual IGF-1 concentrations	3 (6%)	[31, 56, 66]
% Change mean	2 (4%)	[29, 39]
% Change mean [95% CI]	2 (4%)	[29, 41]
Geometric mean [95% CI]	2 (4%)	[40, 41]
% Biochemical control [90% exact CI] Clopper-Pearson exact 2-sided 90% CI	1 (2%)	[37]
% Change mean ± SD [Range]	1 (2%)	[65]
% Change mean [SEM]	1 (2%)	[23]
Geometric mean [68% CI]	1 (2%)	[40]
Mean ± SE [range]	1 (2%)	[58]
Median	1 (2%)	[42]
Time to nadir IGF-1 (mean ± SD)	1 (2%)	[65]
IGF-1 hormone assay reported		
Yes (%)	40 (83%)	[19, 21, 23–36, 38, 40–46, 49, 52–61, 63–67]

*SD-Score* Standard deviation score, *ULN* upper limit of normal, *CI* confidence interval, *SD* standard deviation, *IQR* interquartile range, *SEM* standard error of mean, *SE* standard error

**Table 2:** Overview of the methods, cut-off and statistical reporting for growth hormone (n = 44), ordered by frequency

GH analysis	Number of studies	References
Method		
1 Random sample	10 (23%)	[21, 22, 35, 36, 45, 49, 50, 55, 59, 63]
1 Fasting sample	8 (18%)	[25, 26, 28, 30, 31, 48, 62, 67]
Mean of 5 samples (2 h period)	8 (18%)	[34, 35, 37, 39–43]
Mean of 2/3 fasting samples (15–30 min interval)	1 (2%)	[31]
Mean of 3 fasting samples (1 h interval)	1 (2%)	[23]
Mean of 4 samples (30 min interval)	1 (2%)	[46]
Mean of 4 samples (1 h interval)	1 (2%)	[44]
Mean of 4 samples (4 h interval)	1 (2%)	[48]
Mean of 5 samples (10–15 min interval)	1 (2%)	[29]
Mean of 6 samples (2.5 h period)	1 (2%)	[22]
Mean of 8–10 samples (1 h interval)	1 (2%)	[32]
Not reported	14 (32%)	[19, 38, 47, 51–54, 56–58, 60, 64–66]
GH cut-off for biochemical control		
< 2.5 ng/ml	31 (70%)	[22, 23, 26, 28–30, 32, 34–37, 39–44, 46–51, 53, 56–58, 60, 62, 64, 67]
< 1 ng/ml	16 (36%)	[21, 23, 25, 28, 29, 32, 34, 36, 44, 48, 50, 51, 53, 57, 59, 62]
> 20% decrease from baseline	2 (5%)	[21, 36]
< 1.5 ng/ml	1 (2%)	[45]
< 2 ng/ml	1 (2%)	[19]
< 5 ng/ml	1 (2%)	[19]
> 50% decrease from baseline	1 (2%)	[44]
No cut-off reported/used	8 (18%)	[31, 38, 52, 54, 55, 63, 65, 66]
Summary statistics		
% Biochemical control	31 (70%)	[19, 21–23, 25, 26, 28, 29, 32, 34, 35, 39, 41–51, 53, 56–60, 62, 67]
Mean ± SD	18 (41%)	[23, 29, 34, 35, 39, 41, 42, 44, 46, 48, 54, 55, 57, 62, 63, 65–67]
Individual concentrations	13 (30%)	[25, 30–32, 37, 38, 41, 44, 45, 48, 54, 56, 63]
Median (IQR)	10 (23%)	[21, 28, 29, 34, 45, 48, 49, 51, 53, 59]
% Biochemical control [95% CI] (no method reported)	8 (18%)	[23, 34, 36, 39, 42–44, 46]
% Change from baseline mean + SD	7 (16%)	[22, 35, 46, 48, 57, 63, 67]
Median [range]	7 (16%)	[37, 42, 48, 52, 54, 57, 64]
Mean	4 (9%)	[19, 39, 43, 47]
Mean ± SD [range]	4 (9%)	[30, 47, 54, 60]
% Change from baseline median	3 (7%)	[22, 37, 64]
% Change from baseline median [range]	3 (7%)	[37, 51, 57]
% Change from baseline median (IQR)	3 (7%)	[32, 40, 49]
Mean ± SE	3 (7%)	[29, 36, 43]
% Change from baseline mean	2 (5%)	[29, 39]
% Change from baseline mean (95% CI)	2 (5%)	[29, 41]
Geometric mean [95% CI]	2 (5%)	[40, 41]
Mean [range]	2 (5%)	[25, 58]
Range	2 (5%)	[31, 35]
% Biochemical control [90% exact CI] (Clopper-Pearson exact 2-sided 90% CI)	1 (2%)	[37]
% Change from baseline individual	1 (2%)	[31]
% Change from baseline mean [range]	1 (2%)	[31]
Geometric mean [68% CI]	1 (2%)	[40]
Maximum observed GH concentration	1 (2%)	[26]
Median	1 (2%)	[42]
Proportion above 40 ng/ml	1 (2%)	[26]
Growth hormone assay reported		
Yes (%)	34 (77%)	[19, 21, 23, 25, 26, 28–32, 34–36, 40–46, 48, 49, 52–54, 56–60, 63, 64, 66, 67]

*CI* Confidence interval, *SD* standard deviation, *IQR* interquartile range, *SE* standard error

**Table 3:** Overview of the methods, cut-off and statistical reporting for the oral glucose tolerance test (n = 11), ordered by frequency

OGTT analysis	Number of studies (%)	References
Method		
2 h period: pre-dose, 30, 60, 120 min	1 (9%)	[24]
2 h period: 30, 60, 90, 120 min	1 (9%)	[45]
3 h period: pre-dose, 30, 60, 90, 120, 180 min	1 (9%)	[33]
Not reported	8 (73%)	[27, 30, 38, 59, 61–63, 67]
Glucose administration		
75 g	5 (45%)	[24, 27, 33, 45, 59]
Not defined	6 (55%)	[30, 38, 61–63, 67]
Nadir cut-off for biochemical control		
< 1 ng/ml	5 (45%)	[24, 33, 59, 62, 67]
< 0.4 ng/ml	1 (9%)	[67]
< 1 µg/dl	1 (9%)	[61]
< 2 mU/L	1 (9%)	[27]
No cut-off reported/used	4 (36%)	[30, 38, 45, 63]
Summary statistics		
% Biochemical control	5 (45%)	[24, 27, 33, 61, 62, 67]
Individual levels	3 (27%)	[24, 30, 38]
Mean ± SD	3 (27%)	[24, 33, 63]
Median (IQR)	3 (27%)	[24, 45, 59]
% Nadir change from baseline mean ± SD	1 (9%)	[24]
Mean ± SD [range]	1 (9%)	[30]
Mean ± SD pre-glucose GH	1 (9%)	[24]
Median	1 (9%)	[62]
Median (IQR) pre-glucose GH	1 (9%)	[24]
Growth hormone assay reported		
Yes (%)	7 (64%)	[30, 33, 45, 59, 61, 63, 67]

*SD* Standard deviation, *IQR* interquartile range

**Table 4:** Overview of the studies including a pharmacokinetic analysis. Ordered by drug and date of publication

Author	Number of samples per subject	Drug	Study design	Analysis summary
*Lanreotide*				
Garrido et al. [20]	10	Lanreotide autogel	Phase II, multicenter, randomized in acromegaly patients	Population PK/PD model linking the PK to individual GH (mean of 7 measurements with 30 min interval) and IGF-1 response
Shimatsu et al. [44]	Not reported	Lanreotide	Phase II multicenter, open-label, randomized, parallel-group and phase III open-label, dose-adjustment, long-term treatment	Mean ± SD of C_max_, AUC and C_min_ at sampled time points
Giustina et al. [21]	3	Lanreotide autogel	Prospective, multicenter, randomized, open-label	Graphical analysis of individual serum concentrations and mean of 2 individual serum concentrations versus IGF-1 concentrations, with linear regression
*Octreotide*				
Gadelha et al. [35]	25	Octreotide implant	Phase II, open-label, randomized	Mean ± SD of C_max_, AUC_0−6 months_, t_max_. Graphical analysis of concentrations with mean ± SD
Chieffo et al. [36]	16	Octreotide LAR Octreotide implant	Phase III, open-label, multicenter, randomized	Graphical analysis with mean ± SE at sampled time points per cohort
Melmed et al. [34]	14	Oral octreotide	Phase III, multicenter, open-label, dose-titration	Mean ± SD for the C_0_, AUC and t_1/2_. Graphical analysis showing the mean ± SE
*Pasireotide*				
Petersenn et al. [46]	3 per scheduled visit, with ~ 20 visits per subject	Pasireotide	Open-ended extension of a phase II study	Individual dose normalized C_trough_ concentration–time profiles
Petersenn et al. [22]	16	Pasireotide LAR	Phase I, randomized, multicenter, open-label	Graphical analysis of mean ± SE C_trough_ concentrations over 84 days and mean ± SE of post-first injection day. Median and mean ± SD for the C_max_, C_trough_, AUC and accumulation ratio
*Pegvisomant*				
Higham et al. [66]	2	Pegvisomant	Prospective, multicenter, open-label	Mean ± SD of concentrations at 2 time points

*PK* pharmacokinetics, *PD* pharmacodynamics, *IGF*-1 insulin-like growth factor 1, *SD* standard deviation, *SE* standard error, *C*_*max*_ apparent maximum concentration, *AUC* area under the concentration–time curve, *C*_*min*_ apparent minimal concentration, *C*_*0*_ apparent initial concentration, *C*_*trough*_ apparent concentration before next dosing, *t*_*1/2*_ half-life

### Insulin-like growth factor-1

The majority of studies that reported IGF-1 outcomes included one fasting or one/two random sample(s) for the assessment of the IGF-1 concentrations (54%), others did not report the sampling design. IGF-1 was reported as ULN corrected levels in 60%, and in concentration units (ng/ml) in 44% of the studies. The reporting of both ULN corrected levels and IGF-1 concentrations also occurred. The ULN corrected cut-off used to assess individual biochemical control, ranged from < 1.0x ULN to < 1.5x ULN. The used summary statistics to report the IGF-1 concentrations ranged from individual profiles, geometric means with confidence intervals, % change from baseline, to time of nadir IGF-1 concentrations. The % biochemical control, individual ULN corrected levels and mean ± SD were most commonly used. A total of 22 other ways of reporting the IGF-1 concentrations were identified and 83% of the studies reported the used IGF-1 assay.

### Growth hormone

A wide variability was observed in the sampling schedule used to measure GH, ranging from 1 random sample to the mean of 8–10 samples taken with 1 h intervals. A total of 10 studies used a random 1 point sample whereas 14 studies did not report the number and the timing of samples taken. The most commonly used GH cut-offs were < 1 ng/ml and < 2.5 ng/ml, used in 36 and 70% of studies respectively, indicating that multiple cut-offs were reported in an individual study. In the 44 studies reporting GH results, 25 different ways were used to report the GH summary statistics, with the % biochemical control, individual concentrations and mean ± SD as most prevalent outcomes. A total of 34 out of 45 studies reported the used GH assay.

### Oral glucose tolerance test

Four studies reported the execution of an OGTT in their methods section but did not report any results, these studies were therefore excluded from the summary table [19, 34, 35, 47]. In the majority of the included studies, the used methodology for an OGTT was not reported (8 out of 11). For the studies that did report the methodology, different sampling schedules were used, although all did use a glucose loading of 75 g. The interpretation of the outcomes of the OGTT varied between studies, with cut-offs for GH ranging from 0.4 ng/ml to 1 µg/dl. The majority of studies (n = 6) did not use the OGTT results in determining the biochemical control of a patient and only reported summary statistics or individual GH concentrations. The used GH assay was reported in 7 out of 11 studies.

### Pharmacokinetics

A total of 9 studies took samples for PK analysis of the drug of interest. The data were analyzed using a non-compartmental analysis in all but one article that applied a population PK/PD model [20]. Due to the wide range in the number of samples taken in each study, different ways of reporting were used. Most commonly, the graphical analysis was presented as mean ± standard error (SE) over time. The correlation of an individual’s PK with their response on GH or IGF-1, was only reported in 2 studies.

## Discussion

This review clearly demonstrates that many methods are applied to measure and report on biomarkers in acromegaly research. To improve comparability of results between studies and the determination of optimal treatment in acromegaly, protocols should be more uniform on the biochemical reporting. However, different cut-off values and summary statistics are commonly applied to determine when a patient qualifies as being biochemically controlled, limiting the possibility to include the study results in a meta-analysis.

### Insulin-like growth factor-1

To assess if IGF-1 concentrations decrease to ‘safe’ ranges after treatment, the use of ULN corrected levels should be used as a surrogate for treatment effectiveness. Additionally, the influence of age and sex on IGF-1 concentrations needs to be corrected for to enable comparison within a population and between studies. Unless the study population characteristics are similar (small age range, same sex) the reporting of IGF-1 concentrations that are not adjusted for by age and sex adds limited value. However, we observed that 40% of the studies reported IGF-1 concentrations that were not adjusted by the ULN, precluding reliable comparisons of biochemical control between studies. The healthy population that is used as reference to determine normal IGF-1 concentrations over age and sex may also play a role, however it is hypothesized that differences in large reference populations are small. Also, many of the studies (46%) did not clearly report the number of samples taken to measure the IGF-1 concentrations. However, since the serum IGF-1 concentration is assumed to be relatively stable during the day, this will most likely have a limited effect on the outcome [68]. The response to drug treatment can also be judged on whether the IGF-1 reduction is consistent on multiple occasions during treatment, to assess the day-to-day variability. This approach, using longitudinal IGF-1 data, would require validation compared to the use of a single IGF-1 sample which are being measured at a fixed time period after the start of treatment.

The variability in cut-offs that are currently used to determine biochemical control was also identified by Stalla et al. [69]. They identified that 32% of the respondents of an online survey from 45 countries apply a cut-off of 1.3x ULN and 18% use a cut-off of 1.5x ULN. The results of this study are in line with the used cut-off values identified in this review, with 13% of the studies accepting ULN corrected IGF-1 levels to be < 1.2x ULN, whereas one study used a cut-off of 1.5x ULN.

The high proportion of studies (85%) reporting biochemical control can only be used for the comparison between studies, if identical criteria to assess biochemical control are used. However, a total of seven different cut-offs to determine biochemical control were used. The impact of the approach to determine biochemical control is high, which was exemplified by the use of time weighted averages for IGF-1 compared to the use of only a single measurement at the end of treatment, resulting in different outcomes [70]. The majority of studies (83%) reported the used IGF-1 assay. This reporting is especially important when non-corrected IGF-1 concentrations are reported. For the correct reporting of IGF-1 outcomes, the used method of sampling, the criteria for biochemical control (preferably < 1x ULN), and the % of change from baseline per individual should be presented. If non-corrected IGF-1 concentrations are given, the individual’s age and sex should also be included.

### Growth hormone

The wide range of methods to sample GH and determine biochemical control of GH can influence the results, as was recently shown in a paper that suggested that in patients with active acromegaly, the mean of four samples, sampled with 4 h intervals, reflected an endogenous 24 h GH profile best [12]. Only one trial included in this review used this approach to determine the mean GH level, indicating that this sampling method is rarely used in clinical practice [48].

In 70% of the studies, both the GH and IGF-1 concentration were used to define the individual biochemical control, with a multitude of different cut-off values. This percentage is high when taking into account the cautionary remarks in the guidelines on the use of GH levels to determine biochemical control and the lack of a safe reference range. The wide use of < 1 ng/ml or < 2.5 ng/ml as cut-off value for adequate control of GH precludes reliable comparability of studies. The reporting of the percentage biochemical control using both cut-offs would improve this. For GH reporting, only 77% of the studies specified which GH assay was used. This percentage is low, considering the high variability between assays, and should be made mandatory for all future publications. If more than 1 GH assay was used in a study, between or within patients, it should also be noted whether the same international reference was used or what correction to the data was applied [19]. The method of sampling, the used analytical assay, the distribution of GH concentrations and the % of change from baseline are informative to include in a report. Preferably, the GH observations should not be used to assess the biochemical control and treatment effectiveness, due to the reasons previously discussed.

### Oral glucose tolerance test

The OGTT is commonly performed at study initiation for the confirmation of active acromegaly or to assess surgery success multiple weeks after surgery. Unfortunately, there is limited use in performing an OGTT to determine medical treatment efficacy. However, the GH nadir concentrations that are obtained as the main outcome of an OGTT at study initiation or after surgery reflects an individual’s disease state, which may be a predictor of an individual’s response to treatment. Therefore, a consensus in the reporting of the OGTT results would be appropriate to allow comparison between the responses in different biomarkers. The analysis of the OGTT show that different cut-offs were used for the GH response after an OGTT in which biochemical control was most commonly defined as a GH nadir < 1 ng/ml, which is supported by the Endocrine Society guidelines [8]. Only one study performed and reported the sampling schedule as suggested in literature, a 2 h sampling period with samples every 30 min. The majority (64%) of studies provided sufficient details on the used GH assay. Since GH concentrations are measured in the lower assay regions during an OGTT, inclusion of the details of the GH assay used in the methods section is imperative.

### Pharmacokinetics

In this review, nine studies (18% of total) were available that measured at least 1 PK sample. When these studies were explored, all except one study [20], performed a standard non-compartmental analysis. A non-compartment analysis will generally result in the reporting of summary statistics of the secondary PK parameters (C_max_, t_max_, area under the curve) [71]. Alternatively, individual PK profiles are more informative than these summary statistics. This was also the case when the dosage and dose frequency was altered for Lanreotide Autogel [21]. In this case, the individual PK profiles showed a clear overlap between the two cohorts and a high variability within the groups. This could indicate that the variability in drug exposure between individuals is higher than the exposure differences caused by the alternative dosing regimen.

Despite the importance of individualizing treatment responses, the current focus in literature is predominantly on the identification of a dose–response relationship, which neglects the individual concentrations that are reached in patients. In the investigated studies, the reporting of the time after dose was inconsistent. This may have a significant impact on the observed response, which is depended on the drug concentrations at that time point, and should be included in the reporting.

The discrepancy in drug dose and response between individuals might be caused by the high variability in individual serum/plasma drug concentrations. This high variability is often misrepresented due to the reporting of the mean ± SE in PK profiles [22, 34, 36]. Especially in large populations, the use of standard errors are a poor indicator to assess the level of inter-individual variability [72]. This can be clearly observed in the study by Chieffo et al. [36] in which individual concentrations reached 33 times the mean C_max_, which cannot be clearly observed from the reported figure. In this situation, the use of individual profiles, or a 95% confidence interval, is much more informative to quantify and show the inter-individual variability in the PK over time. Besides the variability in response to treatment due to tumor heterogeneity, the impact of different levels of circulating drug levels are commonly ignored.

The studies that measured the PK of the drug had the unique opportunity to investigate the concentration-effect relationship and explore possible covariates, variables that could explain the inter-individual variability in the PK, while studying a wide range of concentrations in a highly heterogeneous population. This approach was only undertaken by Garrido et al. [20] in the development of a population pharmacodynamic model that included drug response on both the individual mean GH and IGF-1 levels, allowing a more evidence based approach in acromegaly treatment.

### Summary statistics

The most common way of reporting the biomarkers concentrations in the included studies was a mean ± SD, which was reported in 42% of the IGF-1 studies and in 41% of the GH studies. However, as a general rule of thumb, the mean ± SD should only be used for normally distributed data [73]. That GH data is commonly non-normally distributed can be clearly observed from the report by Neggers et al., where the depicted standard deviation would indicate that more than 15% of the data are negative GH concentrations [23]. Many tests for data normality exist (e.g. Shapiro–Wilk, Kolmogorov–Smirnov), which are commonly included in statistical software, and are required to be checked as an assumption for some statistical tests [74]. For non-normally distributed data, the reporting of a median and IQR (25–75% distribution of the data) is advised [73]. If data are non-normally distributed, a Mann–Whitney U test can be applied to assess significant differences between groups. Online Resource 4 contains an extensive checklist of the advised reporting of IGF-1, GH, OGTT and PK results in acromegaly studies.

### Study inclusion criteria

In addition to the variability in the reported outcomes, a wide variability in the study inclusion criteria was identified (Online Resource 3). This patient selection criteria differed between studies on the basis of both the used GH and IGF-1 cut-offs or medical treatment history (e.g. treatment naïve, long term treatment), which may significantly alter the study outcomes. However, the impact of patient selection, and the identification of possible differences between patient groups, cannot yet be quantified due to the differences in the methods used to measure and report GH and IGF-1, as identified in this review.

In conclusion, supplementary to a consensus on the diagnosis and the monitoring of treatment effectiveness in acromegaly, a second consensus on reporting of the results of both prospective and retrospective trials is urgently needed. This uniform reporting should, as a minimum, include the patient inclusion and exclusion criteria, the definition of biochemical control used in a study, the proportion of patients achieving biochemical control after treatment (IGF-1 and/or GH), the percentage of change from baseline, the ULN-corrected levels for IGF-1 concentrations (mean/median depending on data normality), the used sampling design, and preferably, individual results. When GH concentrations are reported, the used analytical assay must be included, with the international reference standard. Additionally, the results of an OGTT or the individual PK profiles can be used to obtain explanatory information on an individual’s response to a drug which can be used as a basis for dose optimization. These recommendations will enhance the inter-study comparison and therewith improve evidence based decision making in acromegaly.

## Electronic supplementary material

Below is the link to the electronic supplementary material.

Supplementary material 1 (PDF 66 KB)

Supplementary material 2 (PDF 141 KB)

Supplementary material 3 (PDF 148 KB)

Supplementary material 4 (PDF 138 KB)
