# Supplementary material for: How are growth hormone and insulin-like growth factor-1 reported as markers for drug effectiveness in clinical acromegaly research? A comprehensive methodologic review
Source: Pituitary. 2018 Mar 31;21(3):310–22. doi: 10.1007/s11102-018-0884-4 (PMC5942341; doi:10.1007/s11102-018-0884-4)
Supplement: Supplementary file 1 — Supplementary material 1 (PDF 66 KB) [file 11102_2018_884_MOESM1_ESM.pdf]

Online Resource 1

How are growth hormone and insulin-like growth factor-1 reported as markers for drug effectiveness in clinical acromegaly research? A comprehensive methodologic review

Pituitary

Michiel J. van Esdonk, Eline J. M. van Zutphen, Ferdinand Roelfsema, Alberto M. Pereira, Piet H. van der Graaf, Nienke R. Biermasz, Jasper Stevens, Jacobus Burggraaf

Corresponding author:
Michiel J. van Esdonk
M.j.van.esdonk@lacdr.leidenuniv.nl
Division of Systems Biomedicine and Pharmacology, Leiden Academic Centre for Drug Research, Leiden University, Leiden, The Netherlands.
Tel: +31 71 527 4284

Full search term resulting in 263 abstracts – on 18-10-2017

(**Lanreotide** [tiab] OR Lanreotide [MeSH Terms] OR Somatuline [tiab] OR Somatulin [tiab] OR Somatulina [tiab]
OR BIM 23014 [tiab] OR BIM23014 [tiab] OR angiopeptin [tiab] OR lanreotidum [tiab] OR lanreotida [tiab]

OR **Octreotide** [MeSH Terms] OR Octreotide [tiab] OR Sandostatin [tiab] **OR Sandostatine [tiab]**
OR SMS 201-995 [tiab] OR 201-995 [tiab] OR 201995 [tiab] OR MYCAPSSA [tiab] OR Octreolin [tiab]
OR Octreotide-LAR [tiab] OR CAM2029 [tiab] OR Longastatin [tiab]

OR **Pasireotide** [tiab] OR Pasireotide [MeSH Terms] OR Signifor [tiab] **OR SOM230 [tiab] OR SOM 230 [tiab]**
OR SOM-230 [tiab] OR SOM230B [tiab]

OR **Bromocriptine** [MeSH Terms] OR Bromocriptine [tiab] OR Parlodel [tiab] OR Bromocriptin [tiab] OR Bromocryptin [tiab] OR Bromoergocriptine [tiab] OR Bromoergocryptine [tiab] OR Bromocriptina [tiab]
OR Bromocriptinum [tiab] OR Ergoset [tiab] OR Bromergocryptine [tiab]

OR **Cabergoline** [tiab] OR Cabergoline [MeSH Terms] OR Dostinex [tiab] OR Cabaser, [tiab] OR Cabaseril, [tiab] OR FCE 21336 [tiab] OR FCE-2133 [tiab]

AND (Acromegaly [MeSH Terms] OR Acromegaly [tiab] OR Somatotropinoma [tiab] OR pituitary adenoma [MeSH Terms] OR pituitary adenoma [tiab])

NOT Prolactinoma [tiab]

NOT (Review [Publication Type] OR Case reports [Publication Type])

AND ("2012/01/01"[Date - Publication] : "2018"[Date - Publication])
