# Supplementary material for: How are growth hormone and insulin-like growth factor-1 reported as markers for drug effectiveness in clinical acromegaly research? A comprehensive methodologic review
Source: Pituitary. 2018 Mar 31;21(3):310–22. doi: 10.1007/s11102-018-0884-4 (PMC5942341; doi:10.1007/s11102-018-0884-4)
Supplement: Supplementary file 2 — Supplementary material 2 (PDF 141 KB) [file 11102_2018_884_MOESM2_ESM.pdf]

# Online Resource 2

How are growth hormone and insulin-like growth factor-1 reported as markers for drug effectiveness in clinical acromegaly research? A comprehensive methodologic review

Pituitary

Michiel J. van Esdonk, Eline J. M. van Zutphen, Ferdinand Roelfsema, Alberto M. Pereira, Piet H. van der Graaf, Nienke R. Biermasz, Jasper Stevens, Jacobus Burggraaf

**Corresponding author:**
Michiel J. van Esdonk
M.j.van.esdonk@lacdr.leidenuniv.nl
Division of Systems Biomedicine and Pharmacology, Leiden Academic Centre for Drug Research, Leiden University, Leiden, The Netherlands.
Tel: +31 71 527 4284

**Data extraction form – systemic review; An overview of study characteristics and method disclosure**

| | |
|---|---|
| Title | |
| Authors | |
| Year | |
| Trial identifier | |
| Study design | Prospective/retrospective/cohort/case-control/RCT |
| Study phase | Phase 1/2/3 – Post approval |
| Sample size | Total number of subjects that were included in the study |
| Compound | Compounds used in the study |
| Patient population | What population was studied |
| Response criteria | Biomarkers that were used to determine the effect:<br>A) GH<br>B) IGF-I<br>C) OGTT |
| GH measurement | Sampling design that was used to measure GH.<br>E.g. random 1-point GH measurement, 3-point mean with 30 minute interval. |

| | |
|---|---|
| | |
| GH measurement time point | Was the clock time, time after dose or fasting state of the subjects disclosed? |
| GH cut-off | GH concentration that was used to establish biochemical control |
| IGF-1 measurement | Sampling design that was used to measure IGF-1.<br>E.g. 1 sample, 2 samples |
| IGF-1 measurement time point | Was the clock time, time after dose or fasting state disclosed? |
| IGF-1 cut-off | IGF-1 concentration that was used to establish biochemical control |
| OGTT method | If OGTT results were included, was the method for the OGTT reported?<br>E.g. glucose dose, sampling frequency, cut-off for nadir. |
| PK | Was PK measured? |
| Number of PK samples per subject | Total blood samples taken from 1 individual |
| PK method | Method used to analyze the pharmacokinetic data |
| Statistics | Used summary statistics per biomarker.<br>E.g. Mean (SD); Median (IQR); Median (Range); ULN corrected IGF-1? Yes/No |
| Assay used | Analytical assay used for GH and IGF-1 analysis, if reported. |
