# Supplementary material for: How are growth hormone and insulin-like growth factor-1 reported as markers for drug effectiveness in clinical acromegaly research? A comprehensive methodologic review
Source: Pituitary. 2018 Mar 31;21(3):310–22. doi: 10.1007/s11102-018-0884-4 (PMC5942341; doi:10.1007/s11102-018-0884-4)
Supplement: Supplementary file 3 — Supplementary material 3 (PDF 148 KB) [file 11102_2018_884_MOESM3_ESM.pdf]

Online Resource 3

How are growth hormone and insulin-like growth factor-1 reported as markers for drug effectiveness in clinical acromegaly research? A comprehensive methodologic review

Pituitary

Michiel J. van Esdonk, Eline J. M. van Zutphen, Ferdinand Roelfsema, Alberto M. Pereira, Piet H. van der Graaf, Nienke R. Biermasz, Jasper Stevens, Jacobus Burggraaf

Corresponding author:
Michiel J. van Esdonk
M.j.van.esdonk@lacdr.leidenuniv.nl
Division of Systems Biomedicine and Pharmacology, Leiden Academic Centre for Drug Research, Leiden University, Leiden, The Netherlands.
Tel: +31 71 527 4284

First Author	Year	Title	Number of patients at study completion	Study design	Investigated drug(s)	Patient population
Young Lee	2017	The efficacy of medical treatment in patients with acromegaly in clinical practice.	89	Retrospective	Cabergoline, Bromocriptine, Octreotide LAR, Lanreotide autogel	All patients in the database who were on medication for more than 3 months and began medical treatment after 2000.
Fahlbusch	2017	Surgical debulking of pituitary adenomas improves responsiveness to octreotide lar in the treatment of acromegaly.	38	Randomized, multicenter	Octreotide LAR	Patients with at least one random GH ≥ 12.5 ng/ml and IGF-1 levels > 1x ULN.
Salvatori	2017	A multicenter, observational study of lanreotide depot/autogel (LAN) in patients with acromegaly in the United States: 2-year experience from the SODA registry.	143	Multicenter, open-label, observational	Lanreotide autogel	Patients treated with lanreotide autogel, who had no known sensitivity to SRLs.
Tahara	2017	Efficacy and safety of long-acting pasireotide in Japanese patients with acromegaly or pituitary gigantism: results from a multicenter, open-label, randomized, phase 2 study.	29	Multicenter, open-label, randomized, phase II	Pasireotide LAR	Patients who were medically naïve, GH nadir > 1 µg/L, GH mean > 5 µg/L with IGF-1 levels > 1x ULN or inadequately controlled patients with mean GH > 2.5 µg/l, and IGF-1 levels > 1.3x ULN.
de Fátima Borges	2017	Treatment of acromegaly patients at the Federal University of Triângulo Mineiro(UFTM): Experience Report.	29	Retrospective	Octreotide, Octreotide LAR, Lanreotide, Cabergoline	All patients in the database.

Giustina	2017	High-Dose and High-Frequency Lanreotide Autogel in Acromegaly: A Randomized, Multicenter Study.	29	Prospective, multicenter, randomized, open-label	Lanreotide autogel	Patients with active acromegaly, receiving octreotide LAR or lanreotide autogel with GH levels ≥ 1 µg/L and/or IGF-1 levels of > 1.2x ULN.
Casagrande	2017	Remission of acromegaly after treatment withdrawal in patients controlled by cabergoline alone or in combination with octreotide: results from a multicenter study.	16	Prospective, multicenter	Withdrawal after cabergoline or combined with octreotide LAR	Patients controlled by combination therapy with octreotide LAR and cabergoline or cabergoline alone (IGF-1 < 1x ULN).
Kasuki	2016	Experience with pegvisomant treatment in acromegaly in a single Brazilian tertiary reference center: efficacy, safety and predictors of response.	27	Retrospective	Pegvisomant, Octreotide LAR, Cabergoline	Patients who were treated with pegvisomant for at least three months.
Khairi	2017	Clinical Outcomes and Self-Reported Symptoms in Patients With Acromegaly: an 8-Year Follow-Up of a Lanreotide Study	6	Longitudinal follow-up	Lanreotide, Cabergoline, Pegvisomant	Patients who were originally enrolled in the Massachusetts General Hospital site of SALSA.
Puig-Domingo	2016	Use of lanreotide in combination with cabergoline or pegvisomant in patients with acromegaly in the clinical practice: The ACROCOMB study.	108	Retrospective, multicenter, observational	Lanreotide autogel, Cabergoline, Pegvisomant	Patients with active acromegaly, treated with lanreotide and cabergoline or pegvisomant when treatment with a single agent did not give adequate control.
Gheorghiu	2016	Beneficial effect of dose escalation and surgical debulking in patients with acromegaly treated with somatostatin analogs in a Romanian tertiary care center.	73	Retrospective	Octreotide LAR, Lanreotide SR	Patients treated with somatostatin analogs.
Casagrande	2017	Long-Term Remission of Acromegaly after Octreotide Withdrawal Is an Uncommon and Frequently Unsustainable Event.	58	Prospective, multicenter	Withdrawal from octreotide LAR	Patients with octreotide LAR for ≥ 24 months, dose, and dose interval unchanged in the last 12 months, mean IGF-1 ≤ 1x ULN and IGF-1 ≤ 1x ULN.
Sagvand	2016	Monotherapy with lanreotide depot for acromegaly: long-term clinical experience in a pituitary center.	63	Retrospective, longitudinal, case-control	Lanreotide depot	Patients receiving lanreotide depot monotherapy continuously for at least 24 months, or surgically cured control patients.
Chang	2016	Serial follow-up of presurgical treatment using pasireotide long-acting release with or without octreotide long-acting release for naïve active acromegaly.	7	Prospective, multicenter, randomized, double-blind Phase III	Pasireotide LAR, Octreotide LAR	Patients with naïve active acromegaly, GH > 5 µg/l and IGF-1 levels > 1x ULN.
Bronstein	2016	Switching patients with acromegaly from octreotide to pasireotide improves biochemical control: crossover extension to a randomized, double-blind, Phase III study.	119	Double-blind, 12 month crossover extension of a phase III trial	Pasireotide LAR, Octreotide LAR	Patients with inadequate biochemical control (GH ≥ 2.5 µg/L and/or IGF-1 > ULN) at the end of core study.
Schmid	2016	Effect of pasireotide on glucose- and growth hormone-related biomarkers in patients with inadequately controlled acromegaly.	198	Prospective, multicenter, randomized,	Pasireotide LAR, Octreotide LAR, Lanreotide LAR	Patients with GH > 2.5 ug/L and IGF-1 levels > 1.3x ULN with octreotide or lanreotide as monotherapy for 6 months or longer.

				parallel-group, phase III		
Neggers	2015	Lanreotide Autogel 120 mg at extended dosing intervals in patients with acromegaly biochemically controlled with octreotide LAR: the LEAD study.	107	Prospective, open-label, non-comparative	Lanreotide autogel, bromocriptine, cabergoline	Patients had treatment with octreotide LAR and had stable doses for ≥ 6 months or 4 months when concomitant dopamine agonist therapy was used.
Shimon	2015	Giant GH-secreting pituitary adenomas: management of rare and aggressive pituitary tumors.	34	Retrospective, multicenter	Octreotide LAR, Lanreotide Autogel, Cabergoline, Pegvisomant	Patients with giant adenomas (adenoma size ≥ 40 mm).
Melmed	2015	Safety and efficacy of oral octreotide in acromegaly: results of a multicenter phase III trial.	82	Multicenter, open-label, dose-titration, baseline-controlled phase III	Oral octreotide capsules	Patients had active acromegaly, with a stable dose of SRLs for at least 3 months. Patients showed a complete or partial response to SRLs, defined as IGF-1 < 1.3x ULN and GH < 2.5 ng/ml.
Vandeva	2015	Treatment outcome results from the Bulgarian Acromegaly Database: adjuvant dopamine agonist therapy is efficient in less than one fifth of non-irradiated patients.	534	Retrospective	Bromocriptine, cabergoline, octreotide LAR, pegvisomant	All patients with at least one relevant medical record.
Evran	2014	Clinical experiences and success rates of acromegaly treatment: the single center results of 62 patients.	62	Retrospective	Octreotide LAR and lanreotide	All patients in the database.
Hatipoglu	2015	Discontinuation of somatostatin analogs while acromegaly is in long-term remission.	16	Prospective	Withdrawal from somatostatin analogs (octreotide, lanreotide)	Patients with stable doses of somatostatin analogs for at least 2 years and in remission (IGF-1 < 1x ULN and GH < 1 ng/ml) for at least 2 years.
Gadelha	2014	Pasireotide versus continued treatment with octreotide or lanreotide in patients with inadequately controlled acromegaly (PAOLA): a randomised, phase 3 trial.	181	Prospective, multicenter, randomized, parallel-group, phase III	Pasireotide LAR, octreotide, lanreotide	Patients with GH > 2.5 µg/L and IGF-1 levels > 1.3x ULN with octreotide or lanreotide as monotherapy for 6 months or longer.
Sheppard	2015	Pasireotide LAR maintains inhibition of GH and IGF-1 in patients with acromegaly for up to 25 months: results from the blinded extension phase of a randomized, double-blind, multicenter, Phase III study.	120	Double-blind, multicenter, extension of a phase III study	Pasireotide LAR, Octreotide LAR	Patients with GH < 2.5 µg/L and IGF-1 levels < 1x ULN at the end of the phase III study were eligible to continue receiving their randomized therapy.
Fougner	2014	Preoperative octreotide treatment of acromegaly: long-term results of a randomised controlled trial.	62	Prospective, randomized, multicenter	Octreotide LAR	Patients were newly diagnosed.
Petersenn	2014	Pharmacokinetics, pharmacodynamics, and safety of pasireotide LAR in patients with acromegaly: a randomized, multicenter, open-label, phase I study.	35	Randomized, multicenter, open-label, phase I	Pasireotide LAR	Patients with IGF-1 levels > 1x ULN and GH nadir ≥ 1 µg/L or mean GH level > 5 µg/l.

Espinosa-de-los-Monteros	2015	Octreotide LAR treatment of acromegaly in "real life": long-term outcome at a tertiary care center.	157	Retrospective	Octreotide LAR	Patients who had not received radiotherapy or concomitant treatment with cabergoline.
Colao	2014	Pasireotide versus octreotide in acromegaly: a head-to-head superiority study.	358	Prospective, randomized, double-blind, multicenter	Pasireotide LAR, octreotide LAR	Patients were treatment naïve de novo or after surgery, with GH > 5 µg/L or GH nadir ≥ 1µg/L and IGF above the ULN.
Caron	2014	Tumor shrinkage with lanreotide Autogel 120 mg as primary therapy in acromegaly: results of a prospective multicenter clinical trial.	64	Prospective, open label, single arm, multicenter, phase IIIb	Lanreotide autogel	Patients were treatment naïve with GH secreting macroadenomas, GH mean or nadir > 1 µg/l and IGF-1 levels above ULN.
Vilar	2014	Can we predict long-term remission after somatostatin analog withdrawal in patients with acromegaly? Results from a multicenter prospective trial.	20	Prospective, multicenter	Withdrawal from octreotide LAR	Patients with two or more years of treatment with octreotide LAR, a stable dose and injection interval every 4 weeks or longer, GH levels < 2.5 ng/ml and normal IGF-1 levels for age, tumor remnant < 10 mm, no radiotherapy and no cabergoline or pegvisomant use over the previous 6 months.
Dias	2013	Acromegaly and pregnancy: a prospective study.	8	Prospective, interventional, multicenter	Withdrawal of octreotide and cabergoline during pregnancy	Pregnant patients with active acromegaly, high level of IGF-1, before pregnancy and available MRI image.
Mangupli	2014	Biochemical and quality of life responses to octreotide-LAR in acromegaly.	28	Retrospective observational	Octreotide LAR	Patients were selected because they had completed at least two quality of life questionnaires.
Chieffo	2013	Efficacy and safety of an octreotide implant in the treatment of patients with acromegaly.	163	Randomized, multicenter, international, open-label, phase III	Octreotide LAR, octreotide implant	Patients with serum IGF-1 > 1.2x ULN, GH nadir ≥ 1.0 ng/ml or confirmation of a GH secreting tumor on pathologic examination of surgically removed tissue, with demonstrated responsiveness to octreotide treatment.
Howlett	2013	Control of growth hormone and IGF1 in patients with acromegaly in the UK: responses to medical treatment with somatostatin analogues and dopamine agonists.	2572	Retrospective database analysis of the UK Acromegaly Register	Somatostatin analogues, dopamine agonists and/or GH antagonist. Not further specified.	All patients in the database.
Sanyal	2012	Outcome in acromegaly: A retrospective analysis.	15	Retrospective	Cabergoline	All patients in the database.
Petersenn	2014	Long-term efficacy and safety of subcutaneous pasireotide in acromegaly: results from an open-ended, multicenter, Phase II extension study.	30	Open-label, open-ended, multicenter extension	Pasireotide	Patients with GH ≤ 2.5 µg/L and normal IGF-1 or that showed clinically relevant improved in the core study.
Annamalai	2013	A comprehensive study of clinical, biochemical, radiological, vascular, cardiac, and sleep parameters in an unselected cohort of patients with acromegaly undergoing presurgical somatostatin receptor ligand therapy.	30	Prospective	Lanreotide autogel	Newly diagnosed, untreated, GH nadir < 0.4 µg/L and IGF-1 > 1x ULN.

Shimatsu	2013	Efficacy, safety, and pharmacokinetics of sustained-release lanreotide (lanreotide Autogel) in Japanese patients with acromegaly or pituitary gigantism.	59	Multicenter, open-label, randomized, parallel-group phase II and an open-label, dose-adjustment, long-term treatment III study	Lanreotide autogel	Patients with active acromegaly with mean serum GH levels > 2.8 ng/ml or > 2.5 ng/ml.
Salvatori	2014	Lanreotide extended-release aqueous-gel formulation, injected by patient, partner or healthcare provider in patients with acromegaly in the United States: 1-year data from the SODA registry.	87	Multicenter observational	Lanreotide depot	Acromegaly patients who are treated with lanreotide depot.
Suda	2013	Efficacy of combined octreotide and cabergoline treatment in patients with acromegaly: a retrospective clinical study and review of the literature.	10	Retrospective	Octreotide LAR, cabergoline	Patients who were treated with octreotide LAR monotherapy for more than 8 months and showed octreotide-resistance.
Velija-Asimi	2012	The efficacy of octreotide LAR in acromegalic patients as primary or secondary therapy.	10	Retrospective	Octreotide LAR	Patients with active acromegaly.
Demir	2012	Improvement in remission rates of the first operation in acromegalic patients.	180	Retrospective	Octreotide	Patients undergone transnasal transsphenoidal adenomectomy at least once.
Gadelha	2012	A subcutaneous octreotide hydrogel implant for the treatment of acromegaly.	45	Two open-label, randomized, multicenter, phase II studies	Hydrated and nonhydrated octreotide implants	Patients with IGF-1 > 1.3x ULN, GH nadir > 1.0 ng/ml with responsiveness to octreotide.<br>Patients with IGF-1 > 1.2x ULN, GH nadir > 1.0 ng/ml, complete or partial responsiveness to a somatostatin analog.
Li	2012	Preoperative lanreotide treatment improves outcome in patients with acromegaly resulting from invasive pituitary macroadenoma.	49	Prospective, randomized	Lanreotide	Newly diagnosed, untreated, GH nadir > 2.5 μg/L, IGF-1 > 1.3x ULN patients with an invasive pituitary macroadenoma.
Bernabeu	2013	Pegvisomant and cabergoline combination therapy in acromegaly.	14	Observational, retrospective, cross-sectional study at 5 tertiary hospitals	Pegvisomant, cabergoline	All patients showed a partial response to maximum doses of long-acting SRL therapy, with IGF-I > 1.2x ULN after a minimum of 6 months of treatment. Before the advent of PEG, some patients had received prolonged treatment with SRL despite showing only a partial response. After SRL therapy, all cases had been on PEG monotherapy.
Higham	2012	Effective combination treatment with cabergoline and low-dose pegvisomant in active acromegaly: a prospective clinical trial.	19	A United Kingdom, five-center, open-label, prospective clinical trial	Pegvisomant, cabergoline	Patients with or without transsphenoidal surgery, with or without radiotherapy, with or without prior medical therapy, with different numbers of pituitary deficiencies.
Ramírez	2012	Discontinuation of octreotide LAR after long term, successful treatment of patients with acromegaly: is it worth trying?	12	Prospective	Withdrawal from octreotide LAR	Patients on octreotide LAR with 2 or more years of treatment, on a stable dose and injection interval of 20 mg every 8 weeks or longer for the previous year, no history of radiation, no cabergoline for the previous 6 months, a GH < 1.5 ng/ml, and an IGF1 < 1.2x ULN.

Tutuncu	2012	Comparison of octreotide LAR and lanreotide autogel as post-operative medical treatment in acromegaly.	68	Retrospective	Octreotide long acting release, lanreotide autogel	Patients not cured by transsphenoidal endoscopic or microscopic pituitary surgery.
Garrido	2012	Pharmacodynamic modeling of the effects of lanreotide Autogel on growth hormone and insulin-like growth factor 1.	104	Phase II, multicenter, randomized	Lanreotide autogel	Patients previously treated or not by surgery, radiotherapy, somatostatin analogues or dopamine agonists.
