# Supplementary material for: How are growth hormone and insulin-like growth factor-1 reported as markers for drug effectiveness in clinical acromegaly research? A comprehensive methodologic review
Source: Pituitary. 2018 Mar 31;21(3):310–22. doi: 10.1007/s11102-018-0884-4 (PMC5942341; doi:10.1007/s11102-018-0884-4)
Supplement: Supplementary file 4 — Supplementary material 4 (PDF 138 KB) [file 11102_2018_884_MOESM4_ESM.pdf]

Online Resource 4

How are growth hormone and insulin-like growth factor-1 reported as markers for drug effectiveness in clinical acromegaly research? A comprehensive methodologic review

Pituitary

Michiel J. van Esdonk, Eline J. M. van Zutphen, Ferdinand Roelfsema, Alberto M. Pereira, Piet H. van der Graaf, Nienke R. Biermasz, Jasper Stevens, Jacobus Burggraaf

Corresponding author:
Michiel J. van Esdonk
M.j.van.esdonk@lacdr.leidenuniv.nl
Division of Systems Biomedicine and Pharmacology, Leiden Academic Centre for Drug Research, Leiden University, Leiden, The Netherlands.
Tel: +31 71 527 4284

Checklist for reporting of acromegaly studies:

1. Insulin-like growth factor 1

- Report method of sampling (single, fasting, mean of multiple samples).
- Report IGF-1 assay, preferably including the international reference standard.
    - Handling of data obtained from 2 or more different assays.
- Report criteria for biochemical control, cut-off for IGF-1?
- Report ULN corrected IGF-1 levels.
- Report % biochemical control and 95%-CI using the pre-defined criteria.
- Report ULN corrected mean ± SD (or median [IQR] if non-normal distribution) of pre-defined subgroups.
- Report % change from baseline with mean ± SD (or median [IQR] if non-normal distribution).
    - Individual baseline and measurements as supplemental material.
        - With an individual’s age and gender when IGF-1 concentrations are given

2. Growth hormone

- Report method of sampling (random, fasting, mean of multiple samples with interval).
- Report GH assay, preferably including the international reference standard.
    - Handling of data obtained from 2 or more different assays.
- Report criteria for biochemical control, cut-off for GH, if any.
- Report % biochemical control and 95%-CI using the pre-defined criteria.
- Report GH mean ± SD (or median [IQR] if non-normal distribution) of pre-defined subgroups.
- Report % change from baseline with mean ± SD (or median [IQR] if non-normal distribution).
    - Individual baseline and measurements as supplemental material.

3. **Oral glucose tolerance test**
   - Report method of sampling and glucose dose.
   - Report GH assay, preferably including the international reference standard.
     - Handling of data obtained from 2 or more different assays.
   - Report GH cut-off for nadir to assess biochemical control, if any.
   - Report % biochemical control and 95%-CI using the pre-defined criteria.
   - Report GH nadir mean ± SD (or median [IQR] if non-normal distribution) of pre-defined subgroups.
   - Report % change from baseline with mean ± SD (or median [IQR] if non-normal distribution).
     - Individual baseline and measurements as supplemental material.

4. **Pharmacokinetics**
   - Report number of samples and time after dosing.
   - Report individual profiles or mean ± 95%-CI to show the inter-individual variability.
   - Report summary and individual statistics from a NCA (C_{max}, t_{max}, AUC, $t_{1/2}$) or, preferably, perform a more advanced statistical analysis (population PK/PD modeling).
   - Link the individual PK to individual response (e.g. linear/non-linear regression of AUC versus ΔIGF-1).
   - Search for and report covariates that explain the inter-individual variability in the PK.
